# Centralized delivery of low-volume biliary biopsies: a novel pretreatment to improve diagnostic accuracy

**DOI:** 10.1055/a-2819-1327

**Published:** 2026-03-16

**Authors:** Bin Yang, Sichao Wen, Haiyong Long, Ping Wang, Wenguang Yang, Yuhong Ren, Mingwen Guo

**Affiliations:** 1 Department of Gastroenterology, Qionglai Medical Center Hospital, Qionglai, China

Peroral cholangiopancreatoscopy (POPS) has been increasingly widely used in clinical practice, including lithotripsy and stone extraction for choledocholithiasis, gallbladder polypectomy, and biliary tract biopsy 1 . Direct visualization-guided biopsy under POPS enables the precise localization of lesions; however, the limited size of biopsy forceps results in a small amount of tissue obtained, which significantly impacts the accuracy of pathological diagnosis. The literature has reported that increasing the number of biopsies or enlarging the diameter of biopsy forceps can improve the biopsy positive rate 2 . But existing methods (e.g., endoscopic retrograde cholangiopancreatography [ERCP] brushing and endoscopic ultrasonography-guided fine needle aspiration/biopsy) still struggle with small sample management, especially in centers without cell-block capacity. Herein, we describe a novel pretreatment method to maximize tissue utilization and enhance positive rates.

**Fig. 1:**
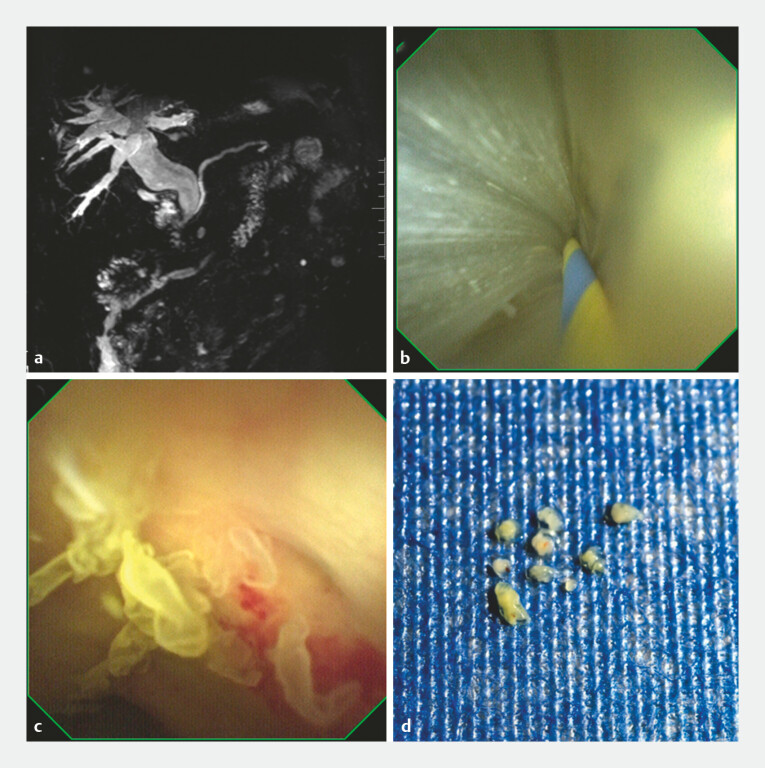
Imaging and endoscopic findings of the biliary tract lesions in the patient. **a** The magnetic resonance imaging (MRI) image showing significant intra- and extrahepatic bile duct dilatation with abnormal signals in the bile duct, suggesting biliary tract pathological changes. **b** A peroral cholangiopancreatoscopy (POPS) view during endoscopic retrograde cholangiopancreatography (ERCP), revealing abundant mucus accumulation in the bile duct. **c** A POPS view displaying multiple papillary translucent lesions on the mucosal surface of the bile duct, which is highly suggestive of biliary neoplastic lesions. **d** A POPS-guided direct visualization biopsy scene, where 10 small biopsy tissue specimens were obtained from the target lesions.

A 76-year-old man presented to our department with scleral and cutaneous jaundice accompanied by anorexia for 1 week. Blood biochemical tests revealed significantly elevated bilirubin levels and predominantly elevated direct bilirubin levels. Magnetic resonance imaging (MRI) showed intra- and extrahepatic bile duct dilatation with abnormal signals in the bile duct ( Fig. 1 **a** ). After obtaining informed consent from the patient, ERCP was performed. Intraoperative POPS examination revealed abundant mucus in the bile duct ( Fig. 1 **b** and Video 1 ) and multiple papillary translucent lesions on the bile duct mucosal surface ( Fig. 1 **c** and Video 1 ). With a clinical diagnosis considered as intraductal papillary mucinous neoplasm of the bile duct (IPMNB), 10 biopsy specimens were obtained under direct POPS visualization ( Fig. 1 **d** and Video 1 ).

Whole-process demonstration of ERCP combined with POPS examination, targeted biopsy, and novel biopsy specimen pretreatment for biliary tract lesions. Final pathological diagnosis: the intraductal papillary mucinous neoplasm of the bile duct (IPMNB). ERCP, endoscopic retrograde cholangiopancreatography; POPS, peroral cholangiopancreatoscopy.Video 1

**Fig. 2:**
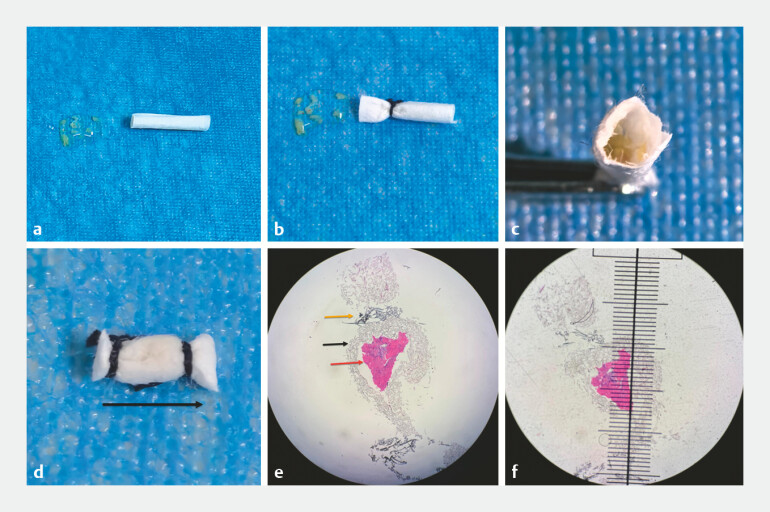
A novel pretreatment process of biliary biopsy specimens and pathological microscopic findings. **a** A small piece of filter paper was cut and shaped into a cylindrical structure for loading biopsy tissues. **b** One end of the filter paper cylinder was tied and sealed with surgical sutures to prevent tissue loss. **c** All 10 biopsy tissue pieces were placed in the prepared filter paper cylinder. **d** After compressing the tissues to aggregate them, the other end of the cylinder was sutured and sealed, with a black arrow marked to indicate the recommended sectioning direction for pathologists. **e** A low-power microscopic view of the pretreated specimen after pathological processing, with the red arrow indicating the aggregated tissue mass, the black arrow indicating the filter paper, and the yellow arrow indicating the sutures. **f** Measurement of the aggregated tissue mass under microscopy, showing a diameter of approximately 1.5 mm, which meets the tissue size requirement for accurate pathological diagnosis.

Our pretreatment protocol was as follows: Step 1: A small piece of filter paper was cut and shaped into a cylindrical form ( Fig. 2 **a** ), with one end tied and sealed using sutures ( Fig. 2 **b** ). Step 2: The biopsy tissue pieces were loaded into the filter paper cylinder ( Fig. 2 **c** ). After compressing the tissue, the other end was also tied and sealed with sutures, and the sectioning direction was indicated to the pathologist (black arrow; Fig. 2 **d** ). After processing by the pathologist, low-power microscopy clearly revealed the tissue mass (red arrow), filter paper (black arrow), and sutures (yellow arrow; Fig. 2 **e** ). The diameter of the aggregated tissue mass was measured to be approximately 1.5 mm ( Fig. 2 **f** ), which met the requirements for pathological diagnosis ( Video 1 ). Final pathological diagnosis was IPMNB.

POPS is widely used for biopsy of biliary tract lesions, but the relatively low biopsy positive rate has long been a challenge for clinicians. Although various countermeasures have been proposed, this issue remains unresolved. Unlike conventional approaches, our method focuses on centralized delivery of multiple small specimens – critical for centers without cell-block capability – avoiding tissue loss and maximizing utilization. We present a novel pretreatment method for biopsy specimens that can effectively improve tissue utilization and thus enhance the positive rate of pathological diagnosis, which is worthy of clinical promotion and application.

Endoscopy_UCTN_Code_CCL_1AZ_2AC Endoscopy_UCTN_Code_TTT_1AR_2AB Endoscopy_UCTN_Code_TTT_1AR_2A
